# A comparative ^18^F-FDG and an anti-PD-L1 probe PET/CT imaging of implant-associated *Staphylococcus aureus* osteomyelitis

**DOI:** 10.3389/fcimb.2023.1182480

**Published:** 2023-05-24

**Authors:** Shu-Qi Ren, Yuan Ma, Li-Lan Fu, Kong-Zhen Hu, Hao-Ran Liang, Bin Yu, Gang-Hua Tang

**Affiliations:** ^1^GuangDong Medical Products Administration (GDMPA) Key Laboratory for Quality Control and Evaluation of Radiopharmaceuticals, Department of Nuclear Medicine, Nanfang Hospital, Southern Medical University, Guangzhou, China; ^2^Guangdong Provincial Key Laboratory of Bone and Cartilage Regenerative Medicine, Division of Orthopedics and Traumatology, Department of Orthopedics, Nanfang Hospital, Southern Medical University, Guangzhou, China

**Keywords:** osteomyelitis, PET imaging, PD-L1, implant-associated *Staphylococcus aureus* osteomyelitis, ^18^F-FDG

## Abstract

**Background:**

Early and accurate diagnosis of infection-induced osteomyelitis, which often involves increased PD-L1 expression, is crucial for better treatment outcomes. Radiolabeled anti-PD-L1 nuclear imaging allows for sensitive and non-invasive whole-body assessments of PD-L1 expression. This study aimed to compare the efficacy of ^18^F-FDG and an ^18^F-labeled PD-L1-binding peptide probe (^18^F-PD-L1P) in PET imaging of implant-associated Staphylococcus aureus osteomyelitis (IAOM).

**Methods:**

In this study, we synthesized an anti-PD-L1 probe and compared its efficacy with ^18^F-FDG and ^18^F-PD-L1P in PET imaging of implant-associated Staphylococcus aureus osteomyelitis (IAOM). The %ID/g ratios (i.e., radioactivity ratios between the infected and non-infected sides) of both probes were evaluated for sensitivity and accuracy in post-infected 7-day tibias and post-infected 21 days, and the intensity of ^18^F-PD-L1P uptake was compared with pathological changes measured by PD-L1 immunohistochemistry (IHC).

**Results:**

Compared with ^18^F-FDG, ^18^F-PDL1P demonstrated higher %ID/g ratios for both post-infected 7-day tibias (P=0.001) and post-infected 21 days (P=0.028). The intensity of ^18^F-PD-L1P uptake reflected the pathological changes of osteomyelitic bones. In comparison to ^18^F-FDG, ^18^F-PDL1P provides earlier and more sensitive detection of osteomyelitis caused by S. aureus.

**Conclusion:**

Our findings suggest that the ^18^F-PDL1P probe is a promising tool for the early and accurate detection of osteomyelitis caused by S. aureus.

## 1 Introduction

Posttraumatic and postoperative osteomyelitis continue to be among the most serious complications following bone trauma or surgery (Odekerken et al., 2014a) Improper treatment of acute osteomyelitis and recurrent episodes of chronic osteomyelitis can lead to limb disability and high amputation rates (Gratz et al., 2001; Conterno and Turchi, 2013). In the early postoperative period, detecting deep orthopedic implant infections can be challenging, making early diagnosis critical for effective treatment and implant survival (Odekerken et al., 2014b). Therefore, having a specific diagnostic tool to monitor implant infections is imperative.

Currently, the early diagnosis of osteomyelitis poses a significant challenge. Among various imaging modalities, such as CT, MRI, labeled leukocyte imaging, and gallium imaging, ^18^F-FDG-PET imaging may play a role in confirming or excluding the diagnosis of peripheral bone osteomyelitis (Makinen et al., 2005; Termaat et al., 2005; van der Bruggen et al., 2010; Lankinen et al., 2012; Chatziioannou et al., 2015; Llewellyn et al., 2019). However, ^18^F-FDG PET has potential limitations as a tool for diagnosing bone infection. This method relies on the intensive glucose consumption of mononuclear cells and granulocytes, which can lead to increased ^18^F-FDG uptake in both bacterial infections and aseptic inflammatory processes (Koort et al., 2004). Because ^18^F-FDG uptake is mediated by metabolism, increased ^18^F-FDG uptake is also associated with acute fractures, normally healing bone and degenerative changes (Koort et al., 2004). Therefore, while ^18^F-FDG-PET imaging may aid in the diagnosis of peripheral bone osteomyelitis, its diagnostic accuracy must be interpreted with caution.

Some studies have indicated that there is a persistent elevation of IFN-γ in bones of mice infected with *S. aureus* by days 3 and 14 post-infection, indicating activation of the immune system and potential bone destruction by these inflammatory factors (Syedbasha and Egli, 2017; Lin et al., 2021). In response to infection, increased levels of proinflammatory cytokines such as IFN-γ and TNF-α can upregulate PD-1/PD-L1 expression locally or systemically (Patil et al., 2018; Curran et al., 2021; Sandker et al., 2022). Furthermore, the detection of increased PD-1/PD-L1 expression in both animal models of *S. aureus* osteomyelitis and human patients with the disease suggests its relevance in the pathogenesis, and at the cellular level, *S. aureus* infection has been demonstrated to induce the expression of PD-1/PD-L1 in bone marrow macrophages (Li et al., 2023). Nuclear imaging, which employs a radiolabeled anti-PD-L1 probe, enables non-invasive, sensitive, and quantitative assessments of PD-L1 expression on a whole-body scale (Niemeijer et al., 2018; Zhou M. et al., 2022). Various preclinical studies in onco-immunology have demonstrated the feasibility of this approach in immunocompetent mouse models (Sun et al., 2022). Moreover, recent clinical studies using a radiolabeled anti-PD-L1 antibody (Bensch et al., 2018), peptide (Zhou X. et al., 2022) demonstrated that nuclear imaging using PD-L1 targeting tracers can assess PD-L1 expression *in vivo*. However, there have been no studies that have conducted an in-depth investigation of PD-L1 expression or imaging in osteomyelitis disease models.

In this study, we have shown that the expression of PD-L1 is upregulated in bone tissues infected with Implant-Associated *S. aureus* osteomyelitis (IAOM) in mouse models. Furthermore, we have identified PET imaging with an ^18^F-labeled PD-L1-Binding Peptide probe (^18^F-PDL1P) as a promising technique for the early detection of bone infections.

## 2 Results

### 2.1 Establishment of IAOM mouse model

**Figure 1:**
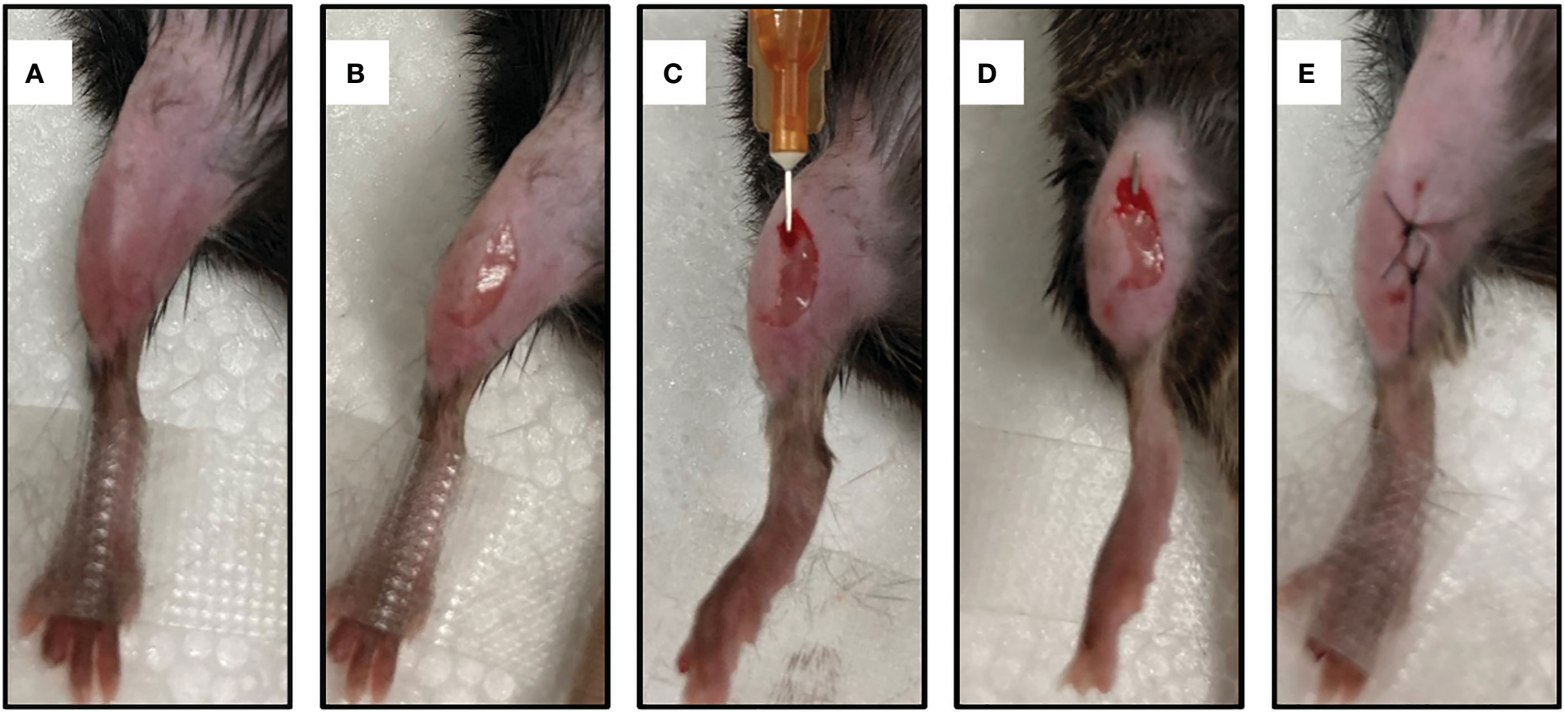
Establishment of a mouse model of implant-associated osteomyelitis. **(A)** skin preparation and disinfection. **(B)** A midline 5mm incision was made with a 15-blade scalpel through the skin at the area of mouse*’*s leg. **(C)** A unicortical defect was made using the needle of a 26-gauge syringe. **(D)** A 9-mm sterile stainless pin (0.5 mm in diameter) was implanted into the medullary cavity. **(E)** The incision was closed with 5-0 silk suture.

To establish a mouse model of IAOM, the hind leg was shaved followed by disinfection with iodine (**Figure 1A**). A 5-mm incision was made on the ventral side of the leg (**Figure 1B**). After the tibia was exposed, a pinhole was drilled by a 26-gauge syringe needle. (**Figure 1C**) Next, a 9-mm ster-ile stainless pin (0.5 mm in diameter) was inserted into the bone marrow cavity through the canal.(For the right side’s infected tibia, implants were soaked in 1* ml* of *S. aureus* solution at 1 × 105 CFU/ml, while the left side’s control received an equal amount of sterile PBS) (**Figure 1D**). The incision was then closed with a 5-0 suture (**Figure 1E**).

### 2.2 Radiographic evaluation and htopathological characteristics of the bone with IAOM

**Figure 2:**
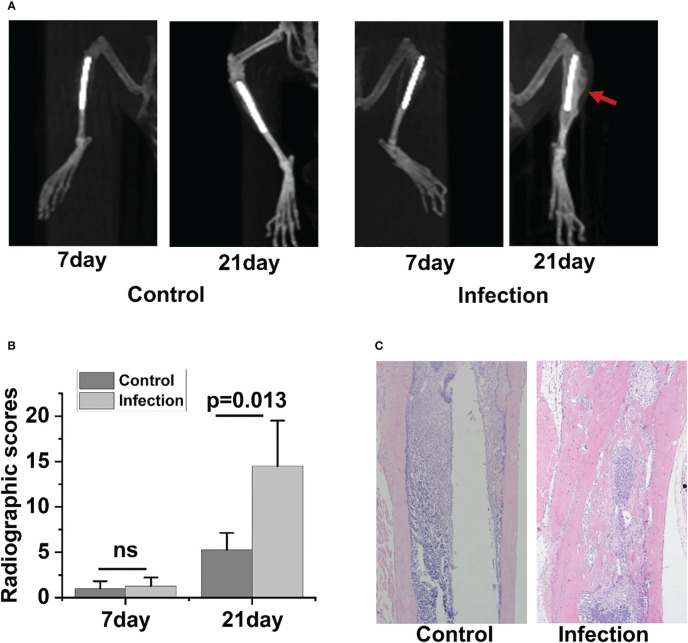
Radiographic and histopathological evidence of bone destruction in infected tibia. Representative radiographic images of tibia bones from control and implant-associated osteomyelitis (IAOM) mice **(A)**. Quantification of bone destruction using a radiographic scoring method **(B)**. Histopathological analysis of tibia in control and infected mice **(C)**. ns, no statistical difference between the two groups at the same time point (n = 4 per group).

Radiographic signs of osteomyelitis were not observed in the uncontaminated control tibia. By day 7 postinfection, there was no discernible change between the infected side and control side in terms of bone shape or intramedullary bone mineral density (BMD)(**Figure 2A**). However, by day 21 post-infection, the infected tibia of IAOM mice exhibited an obvious periosteal reaction, altered bone morphology, increased bone mineral density, and osteolysis around the infected implant, indicating osteonecrosis. Conversely, the implanted bone in the uninfected tibia showed no signs of alteration (**Figure 2A**). Moreover, by day 21 following surgery, the radiographic values for the infected tibia were significantly higher than those for the control tibia (p=0.013) (**Figure 2B**).

To observe the histopathological changes in infected bone, hematoxylin and eosin (H&E) staining was performed in the control and infected tibia on days 21 postoperation. No significant alterations were detected in the bone morphology or histology of the control group. However, as the IAOM progressed to the chronic stage on day 21 post-infection, the *infected* tibia exhibited clear signs of bone destruction, including extensive infiltration of neutrophils in the medullary cavity, the formation of necrotic abscesses in the medullary cavity, deformity of the entire tibia, and sequestrum formation (**Figure 2C**).

In the 3-week follow-up, the severity of radiographic signs of osteomyelitis (periosteal elevation, *cortical* thickening, and osteolysis) was increased in the group of contaminated implants. When the infection occurred 21 days later, the infected tibia showed the typical signs of chronic osteomyelitis. Collectively, the above data demonstrated that the IAOM mouse model showed typical radiologic pathological changes of acute and chronic osteomyelitis along with the time of infection.

### 2.3 Location of *S. aureus* in the bone with IAOM

**Figure 3:**
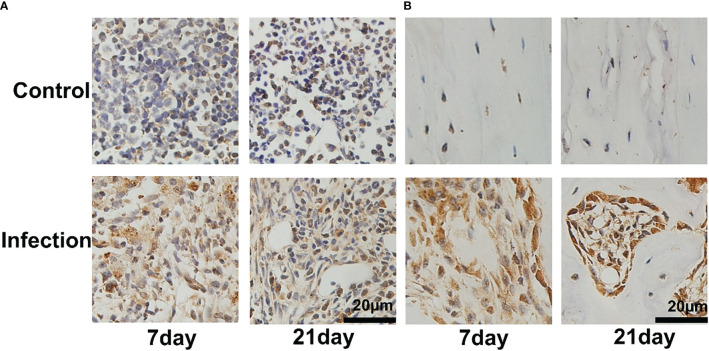
Bacterial colonization of tibial tissue in IAOM mice. Immunohistochemistry images of S. aureus invading and propagating in canaliculi of murine tibiae **(A)** and bone cortex **(B)**. (n = 2 per group). Scale bar =20μm.

To confirm the location of *S. aureus* in infected tibia, immunohistochemical staining was performed. In the infected right tibia, colonization of the bone marrow (**Figure 3A**) and bone cortex (**Figure 3B**) by *S. aureus* was observed at both 7 and 21 days. No colonization of the bone marrow and bone cortex of the left leg tibia by *S. aureus* was observed in the non-*S. aureus*-infected metal graft (**Figure 3A**).

### 2.4 Comparative study of ^18^F-FDG and ^18^F-PDL1P micro-PET imaging

**Figure 4:**
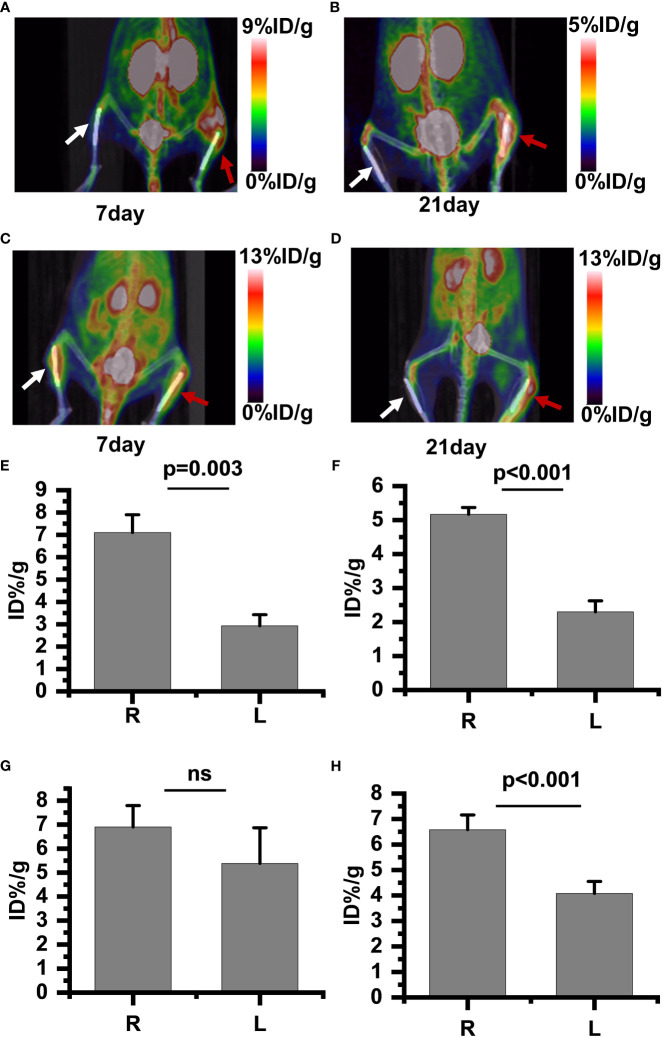
^18^F-PDL1P PET maximal intensity projection (MIP) images of the lower body were taken on post-infection days 7 **(A)** and 21 **(B)**. ^18^F-FDG PET MIP images of the lower body were taken on post-infection days 7 **(C)** and 21 **(D)**. Intake value of ^18^F-PDL1P PET of the post infection on 7 days **(E)** and 21 days **(F)**. Intake value of ^18^F-FDG PET of the post infection on 7 days **(G)** and 21 days **(H)**. The right infected tibia (red arrow) and the left uninfected tibia (white arrow) are shown. ns, no statistical difference between the two groups at the same time point.

PET/CT imaging results revealed a significantly increased uptake of ^18^F-PDL1P in *the infected bone compared to the uninfected tibia during the first week post-surgery* (**Figure 4A**), with no notable increase in ^18^F-FDG uptake (**Figure 4C**). In the subsequent three weeks after surgery, both ^18^F-PDL1P (**Figure 4B**) and ^18^F-FDG (**Figure 4D**) showed an i*n*creased uptake in the osteomyelitic tibia when compared to the opposite uninfected ti*b*ia, as demonstrated by PET imaging.

To quantify the ^18^F-FDG and ^18^F-PDL1P uptake in both groups and investigate whether ^18^F-FDG and^18^F-PDL1P PET enables differentiation between control and infected implants, the uptake of both tracers by the uptake of the bone tissue around the implants was determined. The PET quantitative data demonstrated that during the first week after surgery, there was no significant difference in ^18^F-FDG PET findings between the right and left tibias. Specifically, the %ID/g of the left bone was 5.38 ± 1.66, while that of the right bone was 6.9 ± 0.1 (P=0.117) (**Figure 4G**). However, as osteomyelitis progressed to the third week, the uptake of ^18^F-FDG in the infected bone increased significantly when compared to the contralateral bones (P<0.001) (**Figure 4H**). Notably, the activity of ^18^F-PDL1P was significantly higher in the infected region than in the contralateral bones at both 7 (P<0.003) (**Figure 4E**) and 21 days (P<0.001) (**Figure 4F**).

**Figure 5:**
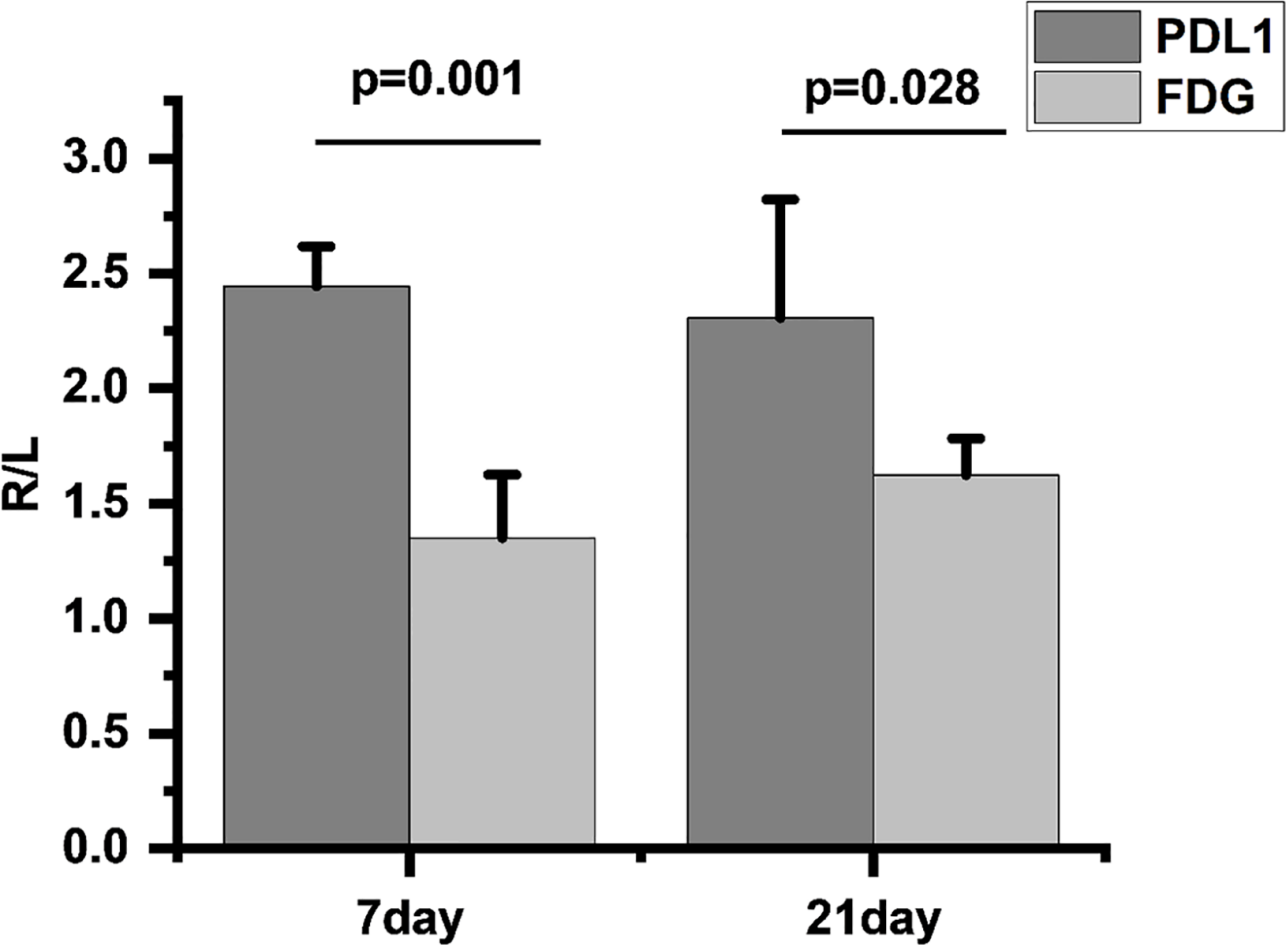
Comparison of ^18^F-FDG and ^18^F-PDL1P uptake rates on infected and uninfected Sides at 7 and 21 Days Post-Infection, R/L(radioactivity ratios between the right infected and left non-infected sides).

We found that compared to ^18^F-FDG, ^18^F-PDL1P showed significantly higher %ID/g ratios in the right (R) and left (L) tibias at both 7 days (P=0.001) and 21 days (P=0.028) post-infection (**Figure 5**).

### 2.5 Locoregional upregulation of PD-L1 in *Staphylococcus aureus* infected bone

To further demonstrate that the accumulation observed in *S. aureus*-infected tibias was predominantly PD-L1 specific, we performed immunohistochemical analysis of the infected tibias.

**Figure 6:**
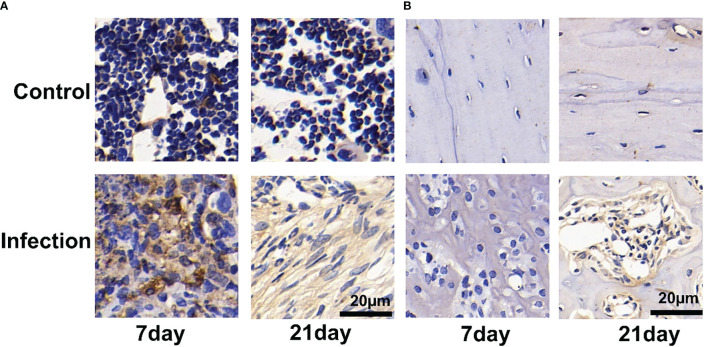
Immunohistochemistry for PD-L1 expression of Staphylococcus aureus-infected and vehicle control implanted bone marrow **(A)** and bone **(B)**. Representative images of immunohistochemistry for PD-L1 in bone and bone marrow (n = 2 per group). Scale bar = 20 μm.

We observed a significant number of PD-L1-positive cells in and around the infection foci in the bone marrow after 7 and 21 days of *S. aureus* infection, but only a few PD-L1-expressing cells were found in the control site (**Figure 6A**). On day 7 post-infection by *S. aureus*, no PD-L1-positive cells were observed in the bone cortex (**Figure 6B**). However, as the infection progressed and worsened at day 21 following infection, we also discovered an expression of PD-L1-positive cells in the bone cortex.

The immunohistochemical analyses show that the uptake of ^18^F-PDL1P in *S. aureus*-infected bone was PD-L1-mediated.

## 3 Discussion

In this experimental study, ^18^F-FDG and ^18^F-PDL1P for PET imaging of bone infection were compared.^18^F-FDG has been adopted as a tracer in PET imaging of bone infections (Koort et al., 2004; Makinen et al., 2005; Lankinen et al., 2012; Odekerken et al., 2014a; Chatziioannou et al., 2015), but the applicability of ^18^F-PDL1P has not been reported for the same indication. By day 7 postinfection, ^18^F-FDG revealed a substantial uptake in uninfected healing-deficient bone and was comparable to infected bone, according to the vivo PET imaging. This led to a false-positive result because it was impossible to discern between infected and uninfected bone. However, during this same time, ^18^F-PDL1P proved useful for distinguishing between infected and uninfected bone. By day 21 postinfection, compared to bones with healing defects, bones with persistent osteomyelitis showed considerably higher uptake of both ^18^F-FDG and ^18^F-PDL1P, according to *in vivo* PET imaging. Furthermore, compared to ^18^F-FDG, ^18^F-PDL1P had a greater rate of increased uptake. The results of our experimental design are consistent with earlier study (Odekerken et al., 2014b) in that the third postoperative week was the earliest time at which it was possible to distinguish between ^18^F-FDG uptake from uninfected healing-deficient bone and infected bone.

*Staphylococcus aureus* remains by far the more common pathogen in osteomyelitis (Chen et al., 2022; Zhang et al., 2022). Clinical data in Nanfang hospital shows that *Staphylococcus aureus* covers 15% of the pathogenic bacteria in osteomyelitis. (*Staphylococcus aureus* covers 15% of the pathogenic bacteria in all surgical site infections (Saadatian-Elahi et al., 2008)). So *Staphylococcus aureus* has the characteristics of stable infection and tissue characteristics close to clinicopathological features (Hidaka, 1985; Liu et al., 2022). Both stainless steel and titanium alloys are the most commonly used materials in orthopedics. There is no significant difference between stainless steel and titanium (Metsemakers et al., 2016), stainless steel implant as infection vectors was used in this study. A well-liked animal model for studying osteomyelitis is the IAOM model of tibial osteomyelitis (Koort et al., 2004; Sun et al., 2022). The model’s great reproducibility in the induction of infection was confirmed by bacteriology. On conventional radiographs and CT scans, as well as histopathologically, the generated bone infection resembled chronic human post-traumatic osteomyelitis. In the current study, the ID/g ratios between the infected and contralateral bones ranged from 1.22 to 1.68 for ^18^F-FDG and from 2.18 to 2.38 for ^18^F-PDL1P, where bacterial osteomyelitis of the mouse tibia was microbiologically verified. Additional studies are necessary to address the following issues: (1) the lack of investigation into the correlation between bacterial load, bacterial activity, and imaging, which could be an essential area for future exploration and may become a focus of our future research direction; and (2) the validation of the diagnostic potential of ^18^F-PDL1P for osteomyelitis using IAOM, as suggested by the findings of this preclinical study, necessitates confirmation through experimentation involving human subjects.

The delicate balancing act between efficient antimicrobial immune defenses and immune-mediated tissue damage appears to be regulated by the PD-1:PD-L1 pathway, which is thought to be a major factor in how an infection will progress (Dyck and Mills, 2017; Qin et al., 2019; Wang et al., 2021; Sandker et al., 2022). Notably, PET imaging of PD-L1 expression has shown to be a reliable predictor of response to immunotherapy and well-correlated with immunohistochemistry (Bensch et al., 2018; Vento et al., 2019; Kelly et al., 2021).Therefore, PET imaging of osteomyelitic using anti-PD-L1P probes is a promising imaging modality. Given that cytokine-induced inflammation can cause a rapid and transient increase in PD-L1 expression levels (Sandker et al., 2022), PD-L1 targeting peptides with shorter circulation times may provide an advantage. With the aid of nuclear imaging, a more strategic design of studies involving immune checkpoint inhibition to treat chronic infectious illnesses complicated by immune dysfunction is now possible. This imaging modality can help assess potential therapeutic synergy between PD-L1 blocking and current antimicrobial immune-activating medications, such as prednisone, TNF-alpha antagonists, and IL-6 blockers, by monitoring changes in PD-L1 expression levels.

Taken together, our findings indicate that^18^F-FDG and ^18^F-PDL1P accumulated in *S. aureus* osteomyelitis. ^18^F-PDL1P in contrast to^18^F-FDG, provides earlier and more sensitive detection of osteomyelitis caused by *S. aureus*. ^18^F-PDL1P was shown to be a potentially effective tracer for the detection of acute osteomyelitic and chronic osteomyelitic with higher T/N(target to nontarget ratio) than ^18^F-FDG.Further studies are needed to clarify the value of ^18^F-PDL1P PET for clinical purposes. Furthermore, our results indicate that ^18^F-PDL1P PET may provide a tool in human clinical diagnostics and for the evaluation of antimicrobial strategies in animal models of orthopedic implant infection.

## 4 Methods

### 4.1 *Staphylococcus aureus* strains and pathogenic challenge

*S. aureus* was isolated from a patient with chronic osteomyelitis, and methicillin-sensitive *S. aureus* was identified using PHOENIX 100 (Becton, Dickinson Microbiology Systems, USA). A frozen stock of *S. aureus* strains was routinely grown on tryptic soy broth (TSB) with shaking at 180 rpm at 37°C for 16 h and collected by centrifugation at 3,000 rpm for 10 min. The bacterial pellets were washed and resuspended in phosphate-buffered saline (PBS). The concentration of *S. aureus* was adjusted to an optical density (OD) of 0.5 at 600 nm, approximately equal to 1 × 10^8^ CFU/ml, and further adjusted to 1 × 10^5^ CFU/ml for soaking implants for IAOM mice.

### 4.2 Implant-associated *S. aureus* osteomyelitis mice model

Protocols for animal experiments were approved by the Animal Care and Use Committee at Nanfang Hospital, Southern Medical University. Male C57BL/6 mice aged 10 to 12 weeks were housed in a facility with a 12-h light/dark cycle, 24 ± 2°C room temperature, and provided with ad libitum access to water and food. The left and right legs of the mice were separately assigned to a self-control group and an IAOM group. Prior to surgery, mice were anesthetized by intraperitoneal injection of tribromoethanol (125 mg/kg of body weight). Implants were soaked in 1 ml of *S. aureus* solution at 1 × 10^5^ CFU/ml for IAOM mice, while an equal volume of sterile PBS was used for the controls. After the hind leg was shaved and disinfected with iodine, a 5-mm incision was made on the ventral side of the leg. The tibia was then exposed, and a pinhole was drilled using a 26-gauge syringe needle. Next, a 9-mm sterile stainless pin (0.5 mm in diameter) was inserted into the bone marrow cavity through the canal. The incision was closed with a 5-0 suture. Both tibiae were collected on days 7 and 21 after the operation for further analysis.

### 4.3 Radiosynthesis

[^18^F]Fluoride was created by bombarding a high pressure [^18^O]H_2_O target with 18 MeV proton beams using a PET trace biomedical cyclotron (PET 800, General Electric, Boston, MA, USA). Radioactivity was measured using a Capintec CAPRAC-R dosage calibrator (NJ, USA). [^18^F]FDG was produced with a specific radioactivity of >76 GBq/mol and a radiochemical purity of >98% using a fully automated FDG synthesis module (IBA). For radiosynthesis of ^18^F-PDL1P, the method described previously was used (Tang et al., 2021; Sun et al., 2022), with manual execution.

### 4.4 PET/CT imaging of IAOM mouse model

Comparative ^18^F-FDG and ^18^F-PDL1P PET/CT imaging was performed at 1 and 3 weeks after a 4-hour fasting period prior to tracer injection. Mice were anesthetized and placed in a micro-PET scanner (Siemens, Erlangen, Germany) in the prone position, followed by tail vein injection of a range of 7.4 MBq to 11.1 MBq of ^18^ F-FDG and ^18^F-PDL1P. Three-dimensional ordered-subset expectation maximum (OSEM) algorithm (Siemens, Erlangen, Germany) was used for image reconstruction with attenuation correction and for anatomical reference with CT data. Images and regions of interest (ROIs) were generated using Inevon Research Workplace 4.1 software (Siemens, Erlangen, Germany). The standardized circular ROI (radius 3.8 mm) of the right operated tibia and the corresponding region of the left contralateral tibia were quantitatively analyzed for ^18^F-FDG and ^18^F-PDL1P uptake, expressed as mean %ID/g. The mean %ID/g was calculated as the mean radioactivity of the ROI divided by the relative injected dose of radioactivity per kilogram of body weight.

### 4.5 Radiographic evaluation and histological analysis

Quantitative evaluation of the IAOM was performed using a modified scale based on previously reported radiographic parameters (Smeltzer et al., 1997). These parameters include periosteal elevation, architectural deformation, widening of the bone shaft, production of new bone, and deformation of soft tissue, which were evaluated as radiographic indicators of disease. Each parameter was scored between 0 and 4, with a score of 4 indicating the most severe evidence of illness. The same researcher (Y.M.), who was blinded to the infection status of each mouse, scored each radiograph. The total score for each sample was calculated as the sum of the scores for the five parameters.

Following infection on day 21, mice were sedated and intracardially given 4% paraformaldehyde. Proximal and middle bone segments of the harvested tibias were preserved in 4% paraformaldehyde overnight at 4°C. The samples were then demineralized in 10% EDTA for ten days, processed, and paraffin-embedded. Subsequently, 4 μm coronal sections were cut and stained with hematoxylin-eosin (H&E)

### 4.6 Immunohistochemistry

After deparaffinization and rehydration, antigen retrieval for immunohistochemical analysis was carried out by incubating the section in a protease K solution (1 mg/ml) at 37°C for 15 min. Endogenous peroxidase activity was then quenched in 3% H_2_O_2_ for 15 min. Sections were treated with the rabbit anti-*S* antibody after being blocked for 1 hour at room temperature with 10% goat serum. Sections were then incubated with mouse anti-PD-L1 antibody (catalog no. MH68942; Abmart, Shanghai, China) or *S. aureus* antibody (catalog no. ab20920; Abcam) for 1 hour at room temperature. Finally, sections were incubated with avidin-conjugated horseradish peroxidase (HRP) complex in accordance with the manufacturer’s protocol (Vectastain ABC HRP kit; Vector Laboratories, USA). In the end, sections’ peroxidase activities were discovered using a kit for 3,3’-diaminobenzidine (DAB) substrate (Vector Laboratories).

### 4.7 Statistical analysis

The standard deviation (SD) and mean were used to express the data. Statistical analysis was performed using SPSS version 22.0 (IBM Corp, Armonk, NY, USA) to assess the significance of differences between two datasets. A p-value less than 0.05 was considered statistically significant.

## 5 Conclusions

In this work, we find that the expression of PD-L1 was increased in the infected bone in mouse models. And PET using an anti-PD-L1 probe is a promising imaging modality for early bone infections.

## Data availability statement

The raw data supporting the conclusions of this article will be made available by the authors, without undue reservation.

## Ethics statement

The animal study was reviewed and approved by Nanfang Hospital animal ethic committee.

## Author contributions

S-QR and YM contribute equally to the work. S-QR: Validation, Formal analysis, Data Analysis, Writing-Review and Editing; YM: Validation, Formal analysis, Resources; L-LF: Writing - Review and Editing; K-ZH: Resources; H-RL: Validation; BY: Project administration; G-HT: Supervision, Funding acquisition, Project administration, Writing - Review and Editing. All authors contributed to the article and approved the submitted version.
